# Enhanced Biosynthesis of Fatty Acids Is Associated with the Acquisition of Ciprofloxacin Resistance in Edwardsiella tarda

**DOI:** 10.1128/mSystems.00694-21

**Published:** 2021-08-24

**Authors:** Yu-bin Su, Su-fang Kuang, Jin-zhou Ye, Jian-jun Tao, Hui Li, Xuan-xian Peng, Bo Peng

**Affiliations:** a Key Laboratory of Functional Protein Research of Guangdong Higher Education Institutes, Department of Biotechnology, College of Life Science and Technology, Jinan University, Guangzhou, China; b Laboratory for Marine Biology and Biotechnology, Qingdao National Laboratory for Marine Science and Technology, Qingdao, China; c Center for Proteomics and Metabolomics, State Key Laboratory of Bio-Control, Guangdong Province Key Laboratory for Pharmaceutical Functional Genes, School of Life Sciences, Sun Yat-sen University, University City, Guangzhou, China; d Southern Marine Science and Engineering Guangdong Laboratory (Zhuhai), Zhuhai, China; Ocean University of China

**Keywords:** *Edwardsiella tarda*, ciprofloxacin resistance, proteomics, energy metabolism, pyruvate cycle, biosynthesis of fatty acids

## Abstract

Misuse and overuse of antibiotics drive the selection and spread of antibiotic-resistant bacteria. Although genetic mutations have been well defined for different types of antibiotic resistance, ways to revert antibiotic resistance are largely unexplored. Here, we adopted a proteomics approach to investigate the mechanism underlying ciprofloxacin resistance in Edwardsiella tarda, a representative pathogen that infects both economic animal species and human beings. By comparing the protein expression profiles of ciprofloxacin-sensitive and -resistant *E. tarda*, a total of 233 proteins of differential abundance were identified, where 53 proteins belong to the functional categories of metabolism, featuring a disrupted pyruvate cycle and decreased energy metabolism but increased fatty acid biosynthesis. The altered pyruvate cycle and energy metabolism were confirmed by gene expression and biochemical assays. Furthermore, the role of fatty acid biosynthesis and quinolone resistance were explored. The expression level and enzymatic activity of acetyl coenzyme A (acetyl-CoA) carboxylase, the first step of fatty acid biosynthesis, were increased in ciprofloxacin-resistant *E. tarda*. Treatment of ciprofloxacin-resistant *E. tarda* with acetyl-CoA carboxylase and 3-oxoacyl-[acyl carrier protein] synthase II inhibitors, 2-aminooxazole and triclosan, respectively, reduced the expression of fatty acid biosynthesis and promoted quinolone-mediated killing efficacy to antibiotic-resistant bacteria. Similar results were obtained in clinically isolated *E. tarda* strains. Our study suggests that energy metabolism has been reprogramed in ciprofloxacin-resistant bacteria that favor the biosynthesis of fatty acid, presenting a novel target to tackle antibiotic-resistant bacteria.

**IMPORTANCE** Edwardsiella tarda is the causative agent of edwardsiellosis, which imposes huge challenges on clinics and aquaculture. Due to the overuse of antibiotics, the emergence and spread of antibiotic-resistant *E. tarda* threaten human health and animal farming. However, the mechanism of ciprofloxacin resistance in *E. tarda* is still lacking. Here, iTRAQ (isobaric tags for relative and absolute quantification)-based proteomics was performed to identify a differential proteome between ciprofloxacin-sensitive and -resistant *E. tarda*. The fluctuated pyruvate cycle and reduced energy metabolism and elevated fatty acid biosynthesis are metabolic signatures of ciprofloxacin resistance. Moreover, inhibition of biosynthesis of fatty acids promotes quinolone-mediated killing efficacy in both lab-evolved and clinically isolated strains. This study reveals that a ciprofloxacin resistance mechanism is mediated by the elevated biosynthesis of fatty acids and the depressed pyruvate metabolism and energy metabolism in *E. tarda*. These findings provide a novel understanding for the ciprofloxacin resistance mechanism in *E. tarda*.

## INTRODUCTION

Edwardsiella tarda is one of the most important pathogens that threaten the sustainability of the aquaculture industry. *E. tarda* infects a broad range of fish species and causes systemic hemorrhagic sepsis, emphysema spoilage disease with swollen skin damage, and ulcers and necrosis of liver, kidney, spleen, and muscle tissue in fish, resulting in enormous economic losses (1–3). In addition, it also infects reptiles, amphibians, birds, mammals, and humans (4, 5).

Besides an immunostimulant-based approach (6–9), antibiotic therapy is still one of the most effective measures to prevent or treat *E. tarda* infection. Among the commonly prescribed antibiotics in aquaculture, quinolones (ciprofloxacin, norfloxacin, and enrofloxacin) are a widely adopted antibiotic class that inhibit a broad spectrum of bacteria with high efficacy and rapid action (10). The bactericidal effect of quinolones is to inhibit both of the type II DNA topoisomerases DNA gyrase in Gram-negative bacteria and topoisomerase IV in Gram-positive bacteria, which are necessary for bacterial DNA replication and repair (11). The uptake of quinolones by Gram-negative bacterial cells is mediated by lipopolysaccharide (LPS) and porin OmpF (12). Quinolones are highly efficient against *E. tarda*, Flavobacterium columnare, Aeromonas hydrophila, *Vibrio* spp., Streptococcus iniae, Streptococcus agalactiae, and other pathogenic bacteria of aquatic animals (13–17).

However, the extensive use of quinolone antibiotics leads to the emergence of drug-resistant bacteria (18, 19), and the numbers of clinically isolated multidrug-resistant strains are increasing (20–22). Furthermore, the horizontal gene transfer of multiple drug resistance genes and intertransmissions of drug resistance genes between microorganisms with direct human association are subjects of huge concern (23, 24). The critical genetic determinants of quinolone resistance are the point mutations at the *gyrA* and *parC* genes, which encode DNA gyrase and topoisomerase IV, respectively. In addition, decreased membrane permeability and elevated efflux pumps are also responsible for quinolone resistance (25). Thus, mechanisms of bacterial resistance to the quinolone antibiotics are multifactorial (26).

Proteomics is an efficient tool to study various complex organisms. iTRAQ (isobaric tags for relative and absolute quantification) labeling in combination with tandem mass spectrometry (MS/MS) is one of the most important tools for qualitative and quantitative protein research (27, 28). The approach has been widely applied to explore mechanisms for antibiotic resistance (29). Carvalhais et al. used iTRAQ to identify differentially expressed proteins in the biofilms Staphylococcus epidermidis that induced and prevented dormancy (30). They demonstrated that the prevented dormancy leads to the expression of significantly different protein sequences compared to dormant biofilms (30). We have shown that the decrease of central carbon and energy metabolisms contributed to the characteristics of Vibrio alginolyticus resistance to levofloxacin (31). We further adopted this technique to investigate the influence of exogenous alanine on global proteome change, and discovered a new mechanism by which alanine promotes reactive oxygen species (ROS) production and facilitates kanamycin to kill antibiotic-resistant bacteria (32). These studies indicate that a proteomics approach can provide additional clues to dissect the mechanism of antibiotic resistance. Therefore, proteomics-based study of more types of the quinolone antibiotics, including ciprofloxacin, is required for further understanding of the multifactorial response.

Here, the iTRAQ technique was adopted to compare differential proteomes between a ciprofloxacin-resistant LTB4 strain (LTB4-R_CIP_) and a ciprofloxacin-sensitive LTB4 strain (LTB4-S). Then, quantitative reverse transcription-PCR (qRT-PCR) and biochemical analysis were used to confirm the data from comparative proteomics. Finally, inhibition of fatty acid biosynthesis sensitized the ciprofloxacin-resistant strain to ciprofloxacin.

## RESULTS

### Identification of proteins using LC-MS/MS combined with iTRAQ labeling.

Bacteria mount proteome-wide responses to antibiotic stress (26). To explore the proteome of ciprofloxacin resistance, iTRAQ-based quantitative proteomics was adopted to identify the protein profiles between 16-fold MICs of LTB4-R_CIP_ and its parental strain, LTB4-S (see Fig. S1A in the supplemental material). Biological replicates of these samples were examined by Pearson correlation, which showed correlation coefficients of 0.81 in LTB4-S and 0.787 in LTB4-R_CIP_ (Fig. S1B and C), indicating that the samples have good reproducibility. Then, a total of 1,913 proteins matched to the protein database were obtained. Compared to LTB4-S, a total of 142 upregulated proteins and 91 downregulated proteins were identified in LTB4-R_CIP_ (see reference 64 and Tables S1 and S2 in the supplemental material). Among these differential proteins, 53 proteins belong to the category metabolism (see Table S3 in the supplemental material). These results suggest that *E. tarda* adopts a proteomic change to fit ciprofloxacin stress.

10.1128/mSystems.00694-21.1 FIG S1 MICs and correlation coefficients between biological replicates of LTB4-S and LTB4-R_CIP_. (A) MICs of LTB4-S and LTB4-R_CIP_. (B) Correlation between all proteins in LTB4-S. (C) Correlation between all proteins in LTB4-R_CIP_. Download FIG S1, TIF file, 1.2 MB. Copyright © 2021 Su et al. 2021 Su et al. https://creativecommons.org/licenses/by/4.0/ This content is distributed under the terms of the Creative Commons Attribution 4.0 International license.

10.1128/mSystems.00694-21.3 TABLE S1 Identification of significantly upregulated proteins involved in *E. tarda* in response of ciprofloxacin resistance using iTRAQ-based proteomics analysis as previously described (64). The proteins with one peptide are identified. Download Table S1, XLS file, 0.07 MB. Copyright © 2021 Su et al. 2021 Su et al. https://creativecommons.org/licenses/by/4.0/ This content is distributed under the terms of the Creative Commons Attribution 4.0 International license.

10.1128/mSystems.00694-21.4 TABLE S2 Identification of significantly downregulated proteins involved in *E. tarda* in response of ciprofloxacin resistance using iTRAQ-based proteomics analysis. Download Table S2, XLS file, 0.05 MB. Copyright © 2021 Su et al. 2021 Su et al. https://creativecommons.org/licenses/by/4.0/ This content is distributed under the terms of the Creative Commons Attribution 4.0 International license.

10.1128/mSystems.00694-21.5 TABLE S3 Identification of significantly differential proteins associated with metabolism in LTB4-R_CIP_ compared to LTB4-S. Download Table S3, XLS file, 0.04 MB. Copyright © 2021 Su et al. 2021 Su et al. https://creativecommons.org/licenses/by/4.0/ This content is distributed under the terms of the Creative Commons Attribution 4.0 International license.

### Protein subcellular localization, molecular function, and biological process.

**FIG 1:**
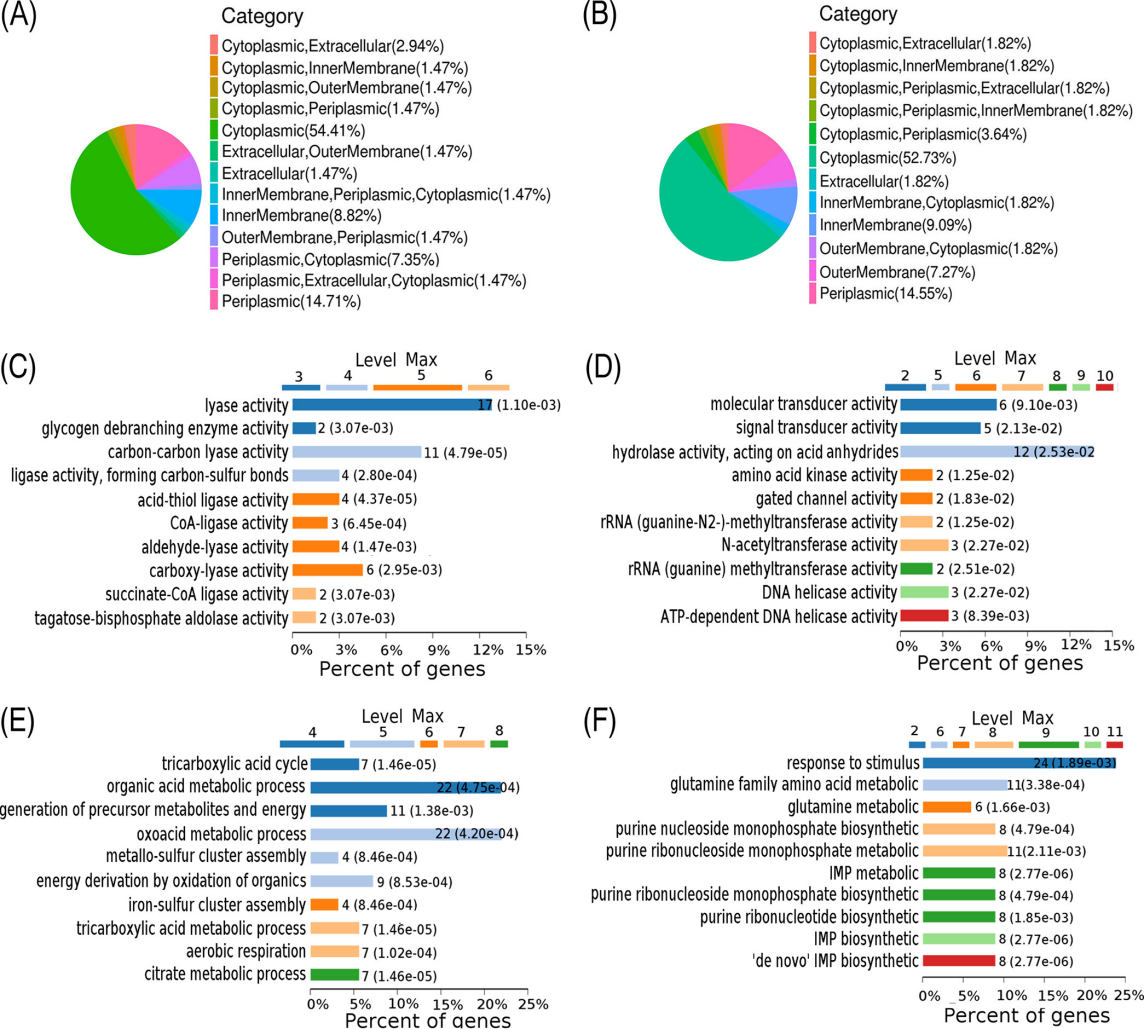
Subcellular localization, molecular analysis, and biological process analysis of differentially expressed proteins. (A and B) The subcellular localization of differentially expressed proteins. (C and D) Molecular functional analysis of differentially expressed proteins. (E and F) Biological process analysis of differentially expressed proteins. Panels A, C, and E show upregulated proteins, and panels B, D, and F show downregulated proteins. “Max” is the maximal annotated level of this term in the GO graph.

Furthermore, differentially subcellular localization of the differential proteins was performed by UniProt database annotation. Among the 142 upregulated proteins and 91 downregulated proteins, more than half of the proteins were localized in cytoplasm (Fig. 1A and B). When molecular function of the differentially expressed proteins was categorized through Gene Ontology (GO) enrichment analysis, 55% of upregulated proteins and 29% of downregulated proteins were enriched in 10 different molecular functions. All of them were involved in enzyme activities, where a majority of the upregulated proteins were ligases or possessing ligase activity, and the downregulated proteins were transducers, transferases, and helicases (some were ATP dependent) (Fig. 1C and D). Moreover, GO analysis showed the biological processes of the differentially expressed proteins. The 142 upregulated proteins were involved in processes related to organic acid metabolism (22%), oxoacid metabolism (22%), generation of precursor metabolites and energy (11%), energy derivation by oxidation of organic compounds (9%), the tricarboxylic acid (TCA) cycle (7%), tricarboxylic acid metabolism (7%), aerobic respiration (7%), citrate metabolism (7%), metallo-sulfur cluster assembly (4%), and iron-sulfur cluster assembly (4%) (Fig. 1E). The 91 downregulated proteins were involved in processes related to response to stimulus (24%), glutamine family amino acid metabolism (11%), purine ribonucleoside monophosphate metabolism (11%), purine nucleoside monophosphate biosynthesis (8%), IMP metabolism (8%), purine ribonucleoside monophosphate biosynthesis (8%), purine ribonucleotide biosynthesis (8%), IMP biosynthesis (8%), *de novo* IMP biosynthesis (8%), and glutamine metabolism (6%) (Fig. 1F). IMP, a key product of purine metabolism, is linked to ATP biosynthesis. The downregulation of the purine and IMP biosynthesis process further indicates that ATP biosynthesis was affected. Therefore, several metabolic pathways are enriched in ciprofloxacin resistance, and the downregulation of energy metabolism is a characteristic feature.

### Protein-protein interaction network analysis and KEGG pathway enrichment.

**FIG 2:**
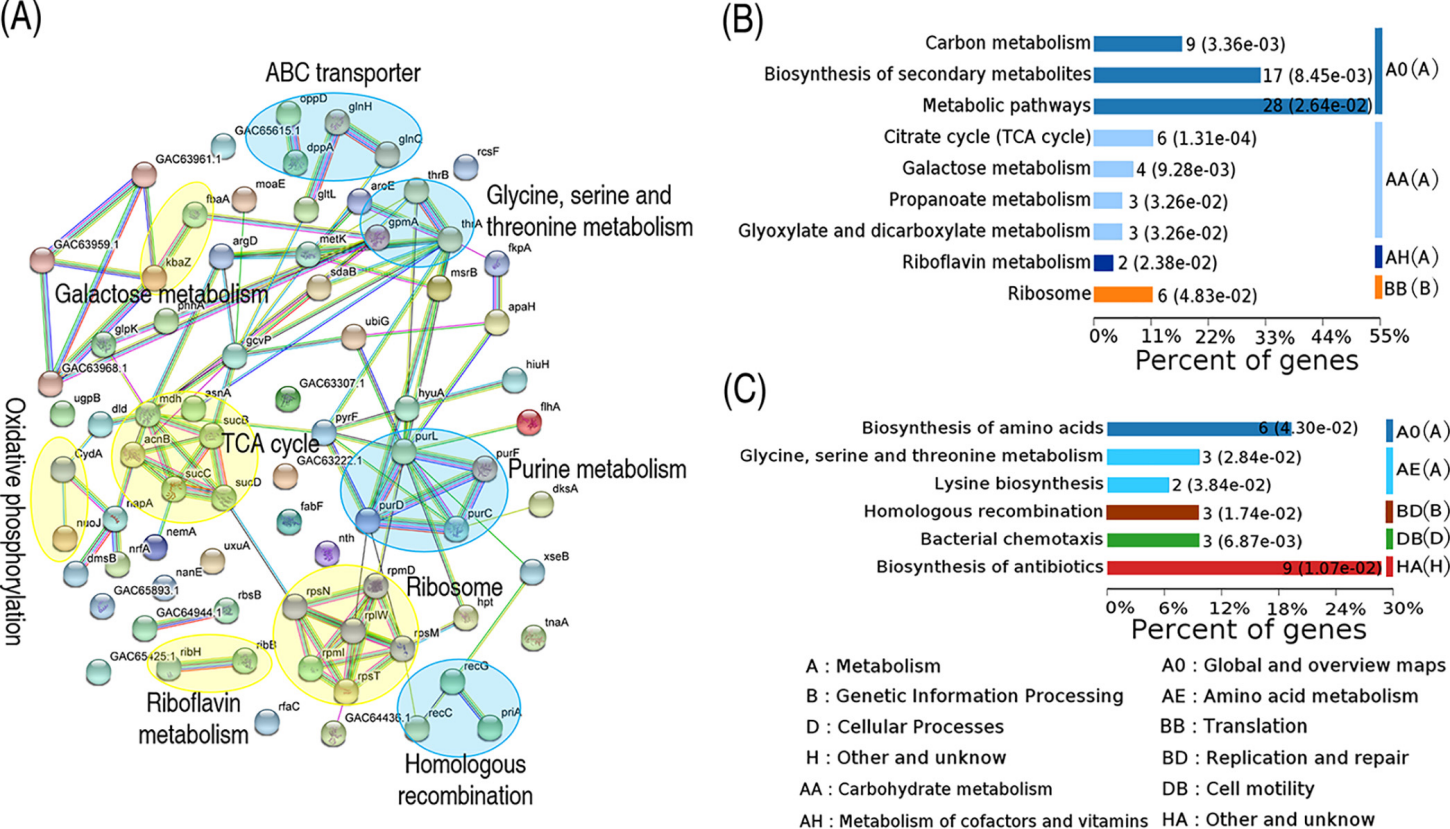
Functional proteins associated with metabolism. (A) Predicted physical and functional network of protein-protein interaction. Yellow, upregulation pathway; blue, downregulation pathway. (B) KEGG function enrichment of upregulation metabolic pathway. (C) KEGG function enrichment of the downregulation pathway.

The protein-protein interaction network is the standard model to describe protein-protein interactions formed by biochemical events and/or electrostatic forces. Hence, we used STRING online software to construct a protein-protein interaction network to explore biochemical characteristics of the ciprofloxacin-resistant proteome. Among the 233 proteins of differential abundance, 60 proteins were matched to the databases. These proteins constituted a complex protein-protein interaction map, in which nine biological functional categories were enriched. Among them, galactose metabolism, TCA cycle, oxidative phosphorylation, riboflavin metabolism, and ribosomes were upregulated, while ABC transporter, glycine, serine and threonine metabolism, purine metabolism, and homologous recombination were downregulated (Fig. 2A). KEGG pathways were further analyzed with OmicsBean (33). The upregulated proteins were enriched to nine pathways, including carbon metabolism, biosynthesis of secondary metabolism, metabolic pathways, the TCA cycle, galactose metabolism, propanoate metabolism, glyoxylate and dicarboxylate metabolism, riboflavin metabolism, and ribosomes (Fig. 2B), whereas, the downregulated proteins were enriched to six pathways, including biosynthesis of amino acids, glycine, serine and threonine metabolism, lysine biosynthesis, homologous recombination, bacterial chemotaxis, and biosynthesis of antibiotics (Fig. 2C). Among the enriched pathways, 4 of the 15 pathways were contributing to the central carbon metabolism and energy metabolism. These results indicate that a protein-protein interaction network is reshaped to cope with ciprofloxacin stress in *E. tarda*, where metabolism plays a key role in this resistance.

### Metabolic network analysis.

**FIG 3:**
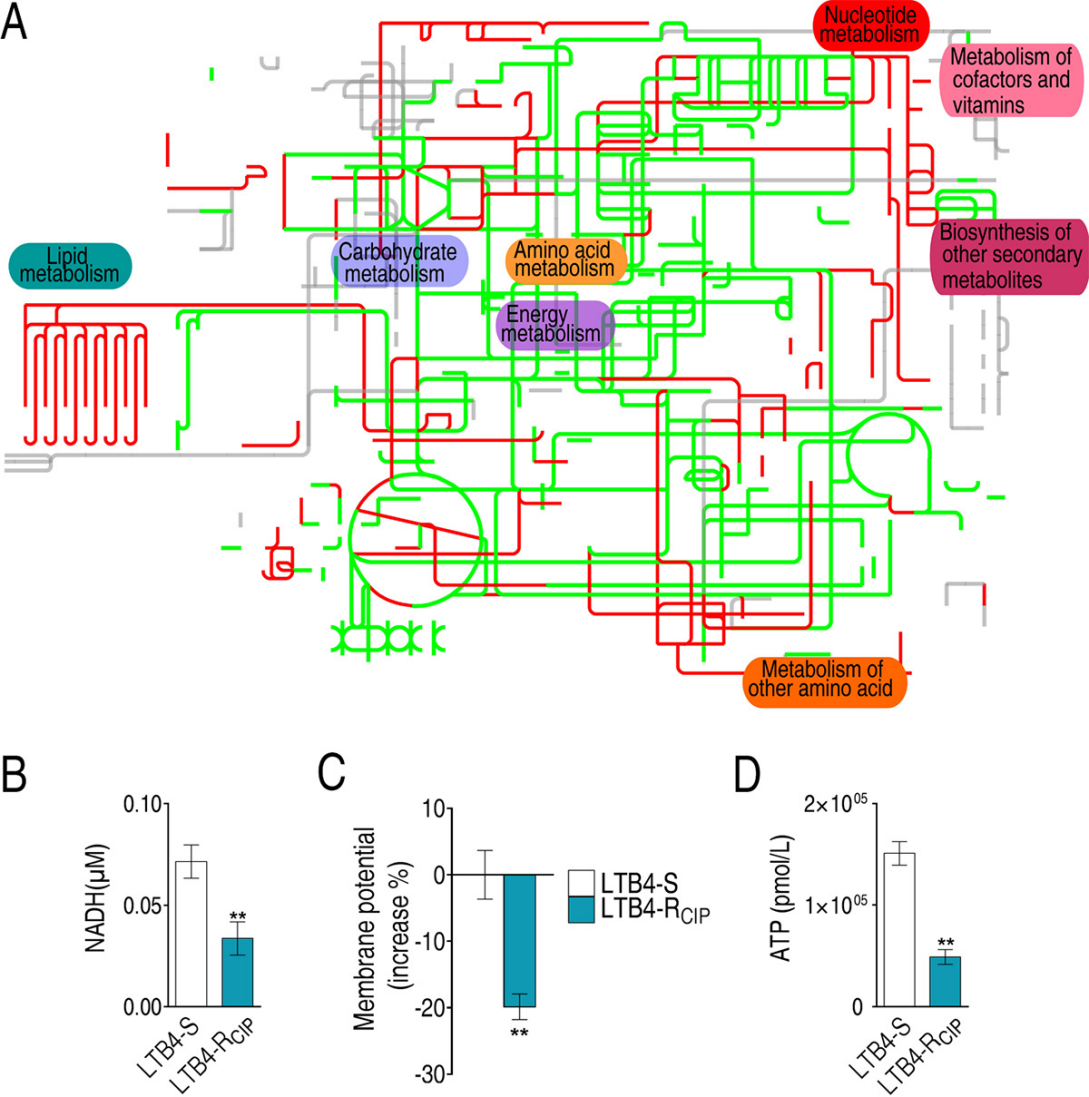
Metabolic pathway analysis. (A) Analysis for metabolic profiling provides better insight into the effects of differential abundance proteins (*P* < 0.05). Based on the eggNOG database (http://eggnog5.embl.de/#/app/home), metabolic network pathways were further analyzed using iPath 3.0 (https://pathways.embl.de/). Red, increase; green, decrease; gray, unaffected. (B to D) Differences in NADH concentration (B), membrane potential (C), and ATP level (D) between LTB4-R_CIP_ and LTB4-S. Results in panels B to D are shown as mean ± standard error of the mean (SEM), and *P* values are identified: *, *P* < 0.05, and **, *P* < 0.01, by two-tailed Student's *t* test.

Since the analysis of the proteome revealed that bacterial resistance was closely related to metabolism, iPath was used to improve the understanding of the global metabolism between LTB4-S and LTB4-R_CIP_, which allows the direct visualization and overview of the biological metabolic pathways. Based on the protein’s eggNOG number, the 233 differential proteins were searched against the iPath database, and 68 proteins were matched. Based on the 68 proteins, a metabolic network was built, where red and green lines indicated upregulated and downregulated pathways in LTB4-R_CIP_, respectively, compared to LTB4-S. Most metabolic pathways, including energy metabolism and the TCA cycle, were downregulated in LTB4-R_CIP_, but lipid metabolism was increased (Fig. 3A). Notably, proteomics analysis did not identify the elevated lipid metabolism, possibly due to there being only two differential proteins detected in the metabolic pathway, but it can be deduced from iPath analysis. Furthermore, iPath showed the inactivation of the TCA cycle, while proteomics characterized the activation of the cycle, which may be related to the fact that only six proteins of the TCA cycle were detected. These data suggest the importance of iPath as an integrated analytic tool for proteomics data. Hence, we speculate that the changes in these metabolisms may affect NADH, membrane potential, and ATP production. Indeed, LTB4-R_CIP_ generated lower levels of NADH, membrane potential, and ATP than those in LTB4-S (Fig. 3B to D). Thus, the results indicate that the TCA cycle and energy metabolism were decreased in response to ciprofloxacin, since NADH, membrane potential, and ATP are generated through the pyruvate cycle (the P cycle) rather than the TCA cycle in bacteria, including *E. tarda* (34). Then, the role of the P cycle in the role of ciprofloxacin resistance was investigated.

### Fluctuation of the P cycle.

**FIG 4:**
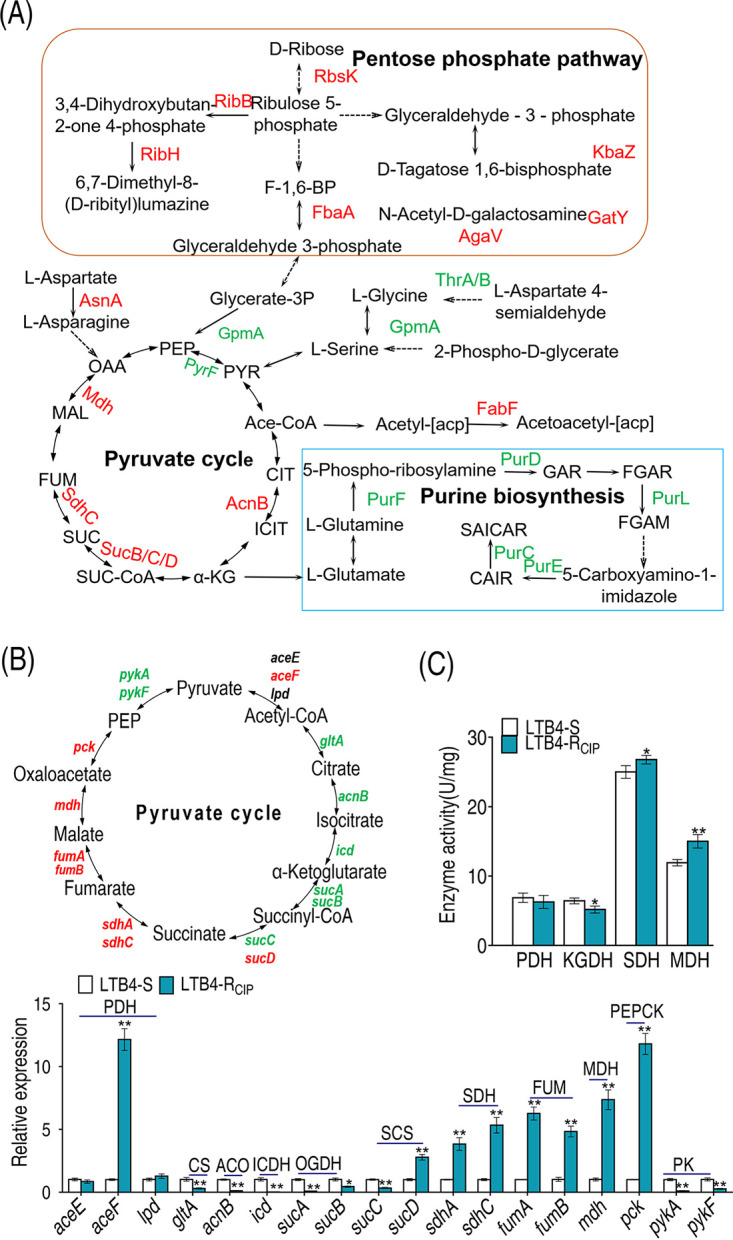
Analysis of P cycle. (A) Analysis of pathways from proteome data. (B) qRT-PCR analysis of P cycle genes. (C) Activities of PDH, KGDH, SDH, and MDH in the P cycle. The upregulated proteins or genes (in red) and downregulated proteins or genes (in green) are all shown in the flowchart. Results in panels B and C are displayed as mean ± SEM, and *P* values are identified: *, *P* < 0.05, and **, *P* < 0.01, by two-tailed Student's *t* test. PDH, pyruvate dehydrogenase; KGDH, α-ketoglutarate dehydrogenase; SDH, succinic dehydrogenase; MDH, malate dehydrogenase.

Based on the proteomics data, the abundance of GpmA and PyrF was decreased: GpmA and PyrF transfer glycerate-3-phosphate to phosphoenolpyruvate (PEP) and PEP to pyruvate, respectively. Although the levels of expression of AcnB, SucB/S/D, SdhC, and Mdh in the P cycle were increased, the decreased expression of GpmA and PyrF was still able to impair the normal function of the P cycle, which was further supported by the reduced purine biosynthesis (reduction of PurF, PurD, PurL, PurE, PurC, and ArgD) (Fig. 4A). Furthermore, qRT-PCR was used to quantify the expression of genes in the P cycle. The majority of the genes were downregulated from PEP to succinate, while all genes were upregulated from succinate to PEP (Fig. 4B). This was consistent with the above data on the decreased purine biosynthesis. Finally, the activities of pyruvate dehydrogenase (PDH), α-ketoglutarate dehydrogenase (α-KGDH), succinate dehydrogenase (SDH), and malate dehydrogenase (MDH) in the P cycle were measured. The activities of SDH and MDH were increased, while the activities of PDH and KGDH were reduced (Fig. 4C), where the reduced activity of PDH was consistent with the decreased expression of *pyrF*. These results indicate that the P cycle fluctuates in resistance to ciprofloxacin.

### Elevation of fatty acid biosynthesis.

**FIG 5:**
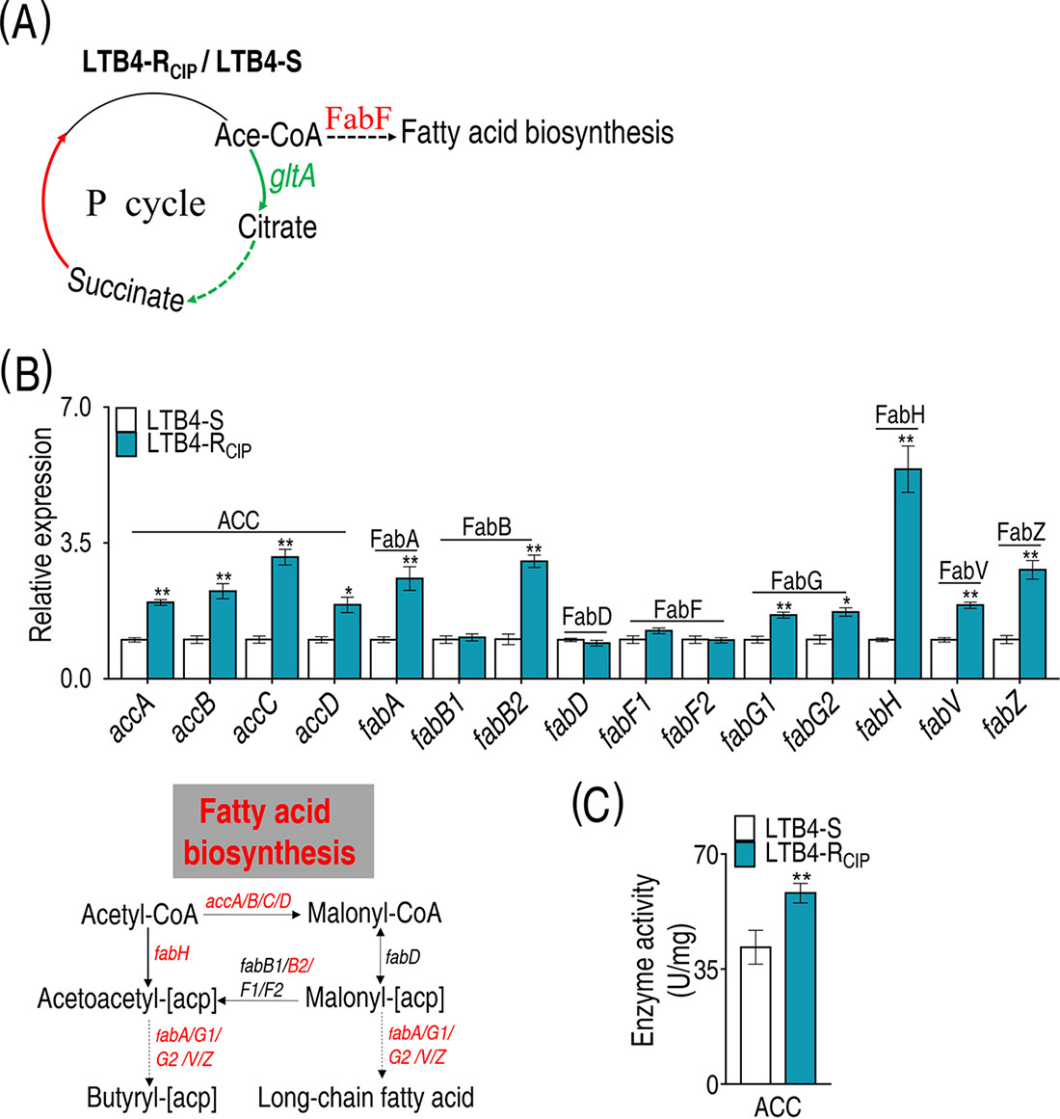
Analysis of biosynthesis of fatty acids. (A) Analysis of pathways from proteome and gene expression data. (B) qRT-PCR analysis of genes for biosynthesis of fatty acids. (C) Activity of ACC in the biosynthesis of fatty acids. The upregulated proteins or genes (in red) and downregulated proteins or genes (in green) are all shown in the flowchart. Results in panels B and C are displayed as mean ± SEM, and *P* values are identified: *, *P* < 0.05, and **, *P* < 0.01, by two-tailed Student's *t* test. ACC, acetyl-CoA carboxylase; FabA, 3-hydroxyacyl-[acyl-carrier protein] dehydratase; FabB, 3-oxoacyl-[acyl-carrier protein] synthase I; FabD, [acyl-carrier protein] *S*-malonyltransferase; FabF, 3-oxoacyl-[acyl-carrier protein] synthase II; FabG, 3-oxoacyl-[acyl-carrier protein] reductase; FabH, 3-oxoacyl-[acyl-carrier protein] synthase III; FabV, enoyl-[acyl-carrier protein] reductase; FabZ, 3-hydroxyacyl-[acyl-carrier protein] dehydratase.

The above results suggested that biosynthesis of fatty acids was enhanced since the expression of *gltA* promoting metabolic flux from acetyl-CoA to citrate was decreased; the expression of FabF catalyzing acetyl acyl carrier protein (acetyl-[acp]) to acetoacetyl-[acp] in biosynthesis of fatty acids was increased (Fig. 5A). To test this, qRT-PCR was used to quantify the expression of genes of fatty acid biosynthesis. Among them, the expression of 11 genes was elevated, while 4 genes, including *fabB1*, *fabD*, and *fabF1*/*F2*, were not changed (Fig. 5B). *fabB2* and *fabB1* encode the two FabB subunits, respectively. Differential expression of different genes encoding subunits of an enzyme has been reported in response to antibiotic stress (35, 36), but the activity of the enzyme is still elevated (36). Thus, the unaffected expression of *fabB1* may not have impacted the activity, but the increased expression of *fabB2* did. *fabD* and *fabF1*/*F2* encode FabD and FabF, respectively, transferring malonyl coenzyme A (malonyl-CoA) to malonyl-[acp] and malonyl-[acp] to acetoacetyl-[acp]. Expression of these genes was not affected, suggesting that acetyl-CoA enhances the metabolic fluxes to butyryl-[acp] but not to long-chain fatty acid synthesis. Acetyl-CoA carboxylase (ACC) is a complex multifunctional enzyme system that catalyzes the carboxylation of acetyl-CoA to malonyl-CoA, the rate-limiting step in fatty acid biosynthesis. LTB4-R_CIP_ had a higher level of ACC activity than LTB4-S (Fig. 5C). These results support the conclusion that biosynthesis of fatty acids is increased in LTB4-R_CIP_.

### Inhibition of fatty acid biosynthesis promotes antibiotic-mediated killing.

**FIG 6:**
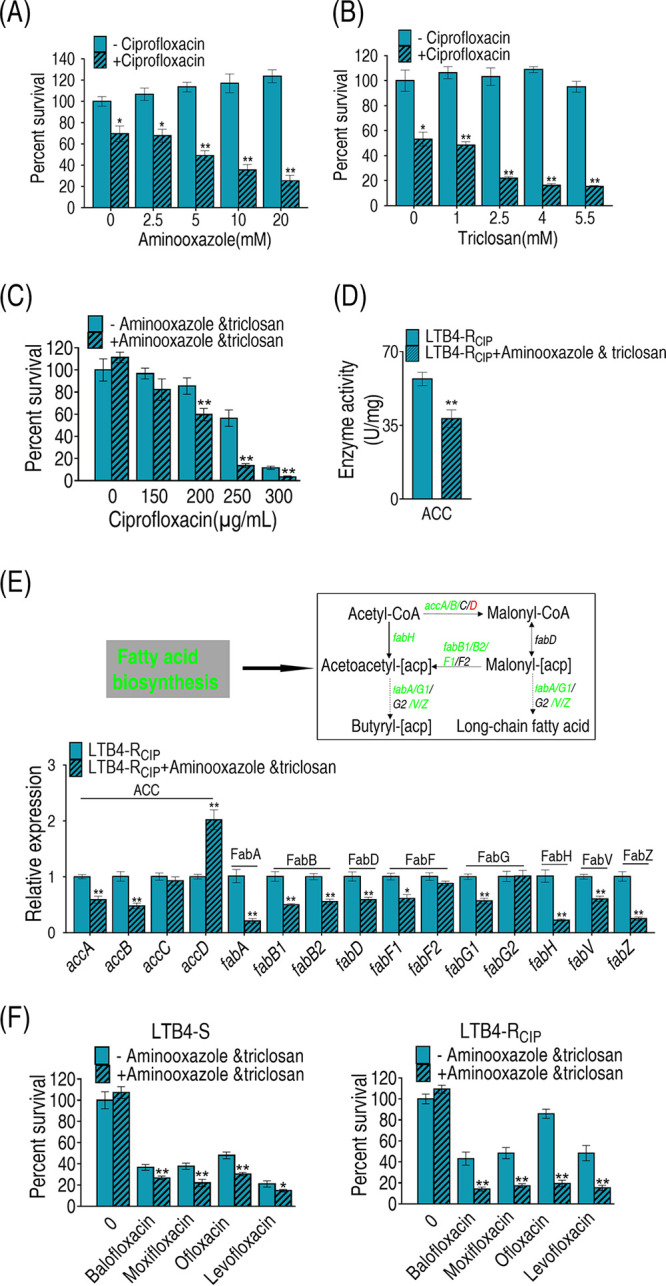
Analysis of biosynthesis of fatty acids in the presence of inhibitors. (A and B) Percentage of survival of LTB4-R_CIP_ in the presence of the indicated concentrations of 2-aminooxazole or triclosan plus 200 μg/ml ciprofloxacin (A) and 250 μg/ml ciprofloxacin (B). (C) Percentage of survival of LTB4-R_CIP_ in the presence of the indicated concentrations of ciprofloxacin plus 20 mM 2-aminooxazole and 4 μg/ml triclosan. (D) Activity of ACC in the presence of 250 μg/ml ciprofloxacin plus 20 mM 2-aminooxazole and 4 μg/ml triclosan. (E) qRT-PCR analysis of genes for biosynthesis of fatty acids with 20 mM 2-aminooxazole and 4 μg/ml triclosan. The upregulated genes are in red, and downregulated genes are in green. (F) Percentage of survival of LTB4-S and LTB4-R_CIP_ in the presence of all kinds of quinolone antibiotics plus 2-aminooxazole and triclosan. Results in panels A to F are reported as mean ± SEM, and *P* values are identified: *, *P* < 0.05, and **, *P* < 0.01, by two-tailed Student's *t* test.

To explore whether the enhanced biosynthesis of fatty acids contributed to ciprofloxacin resistance, two inhibitors of fatty acid biosynthesis, triclosan and 2-aminooxazole, were used. Triclosan inhibits enoyl-ACP reductase (FabI), which catalyzes acyl-[acp] into biosynthesis of saturated fatty acids (37), while 2-aminooxazole selectively targets acetyl-CoA carboxylase (ACC), which catalyzes the first committed step in fatty acid biosynthesis (38). The doses of the inhibitors were chosen by setting up a dose-dependent experiment, where 20 mM 2-aminooxazole achieved the best synergistic effects with ciprofloxacin and was not toxic, while 4 μg/ml triclosan was used, since a concentration higher than that was toxic and caused the death of 10% of the bacteria. Both inhibitors promoted ciprofloxacin-mediated killing. However, the combination of the two inhibitors exhibited higher efficacy than either of the inhibitors alone (Fig. 6C). Consistently, the synergy reduced activity of ACC and expression of genes in most of fatty acid biosynthesis genes (Fig. 6D and E). Specifically, among the above 15 genes of biosynthesis of fatty acids, the levels of expression of 11 genes were decreased and that of 1 gene was increased when inhibitors were present (Fig. 6E). Similar results were obtained with other quinolone antibiotics: balofloxacin, levofloxacin, ofloxacin, and moxifloxacin (Fig. 6F). These results indicate that the elevated biosynthesis of fatty acids serves for quinolone resistance in *E. tarda*.

### Inhibition of fatty acid biosynthesis confers sensitivity of clinical *E. tarda* strains to ciprofloxacin.

**FIG 7:**
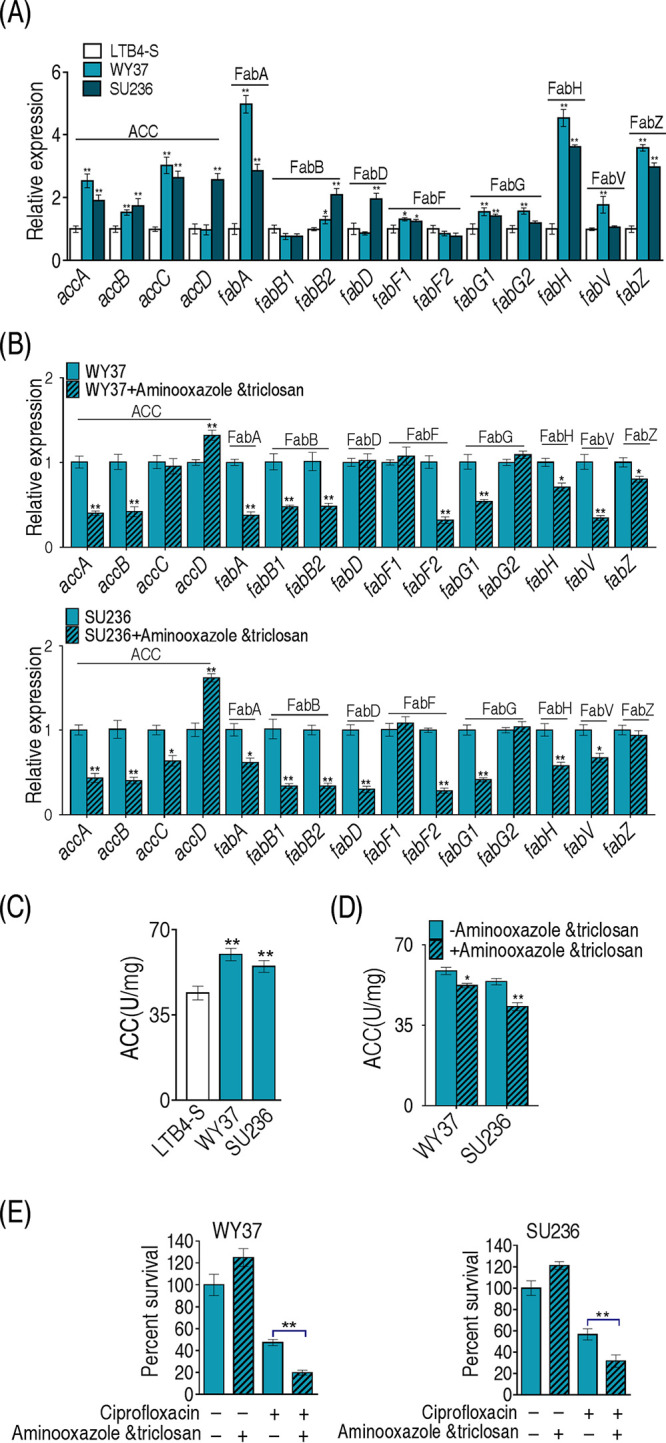
Effects of inhibitors on biosynthesis of fatty acids in clinically isolated *E. tarda* strains. (A) qRT-PCR analysis of genes for biosynthesis of fatty acids between LTB4-S and clinical isolates of *E. tarda*. (B) qRT-PCR analysis of genes for biosynthesis of fatty acids among clinical isolates of *E. tarda* with 20 mM 2-aminooxazole and 4 μg/ml triclosan. (C) Comparison of the activity of ACC. (D) Effects of the addition of 20 mM 2-aminooxazole and 4 μg/ml triclosan on the ACC activity of clinical isolates of *E. tarda*. (E) 2-Aminooxazole and triclosan and ciprofloxacin kill the clinically isolated *E. tarda* strains. Results in panels A to E are displayed as mean ± SEM, and *P* values are identified: *, *P* < 0.05, and **, *P* < 0.01, by two-tailed Student's *t* test.

To test whether the above finding was applicable to clinical *E. tarda* strains, clinically multidrug-resistant *E. tarda* strains WY37 and SU236, which were resistant to at least three classes of antibiotics, including quinolones, were tested (see Fig. S2A in the supplemental material). The growth curve showed that LTB4-R_CIP_ and WY37 grew slower than LTB4-S in the first 8 h, while similar growth was seen between SU236 and LTB4-S (Fig. S2B). The increase of efflux pump expression leads to the reduction of the influx of many antibiotics and resistance to bacteria. As an example, there is the increased efflux of AcrAB-TolC, an energy-dependent efflux system encoded by *acrAB-tolC*, which can excrete intracellular antibiotics extracellularly and thus reduce drug sensitivity (39). However, Western blotting demonstrated that the expression levels of TolC were similar among the four strains (Fig. S2C), and meanwhile, the expression of TolC of ciprofloxacin-resistant *E. tarda* had not changed. This result suggests that other factors, more than TolC, were involved in resistance to quinolones in *E. tarda*. However, a point mutation that occurred on codon 83 (S83R) of the *gyrA* gene was identified both in WY37 and LTB4-R_CIP_ (see Table S4). Furthermore, qRT-PCR was used to quantify the expression of the 15 genes in biosynthesis of fatty acids. Among them, the expression of 11 genes was elevated, and that of 4 genes was unchanged in both WY37 and SU236 (Fig. 7A). These results were similar in LTB4-R_CIP_ (Fig. 5B). Expression of these genes was reduced when treated with the inhibitors 2-aminooxazole and triclosan (Fig. 7B). Activity of ACC was increased in WY37 and SU236 (Fig. 7C), which was inhibited in the presence of 2-aminooxazole and triclosan (Fig. 7D). Consistently, viability of the two clinically multidrug-resistant strains was reduced when the two inhibitors were present (Fig. 7E). These results indicate that biosynthesis of fatty acids contributes to quinolone resistance in clinically multidrug-resistant *E. tarda* strains.

10.1128/mSystems.00694-21.2 FIG S2 Comparison of MICs, Western blot results, and growth curves of six strains of *E. tarda*. (A) MICs of six strains of *E. tarda*. (B) Western blot for detection of TolC. (C) OD values of six strains of *E. tarda* in LB medium at the indicated times. Download FIG S2, TIF file, 1.4 MB. Copyright © 2021 Su et al. 2021 Su et al. https://creativecommons.org/licenses/by/4.0/ This content is distributed under the terms of the Creative Commons Attribution 4.0 International license.

10.1128/mSystems.00694-21.6 TABLE S4 Mutation in the *gyrA* gene. Download Table S4, XLS file, 0.03 MB. Copyright © 2021 Su et al. 2021 Su et al. https://creativecommons.org/licenses/by/4.0/ This content is distributed under the terms of the Creative Commons Attribution 4.0 International license.

### Cell membrane permeability is altered in antibiotic-resistant bacteria with increased fatty acid biosynthesis.

**FIG 8:**
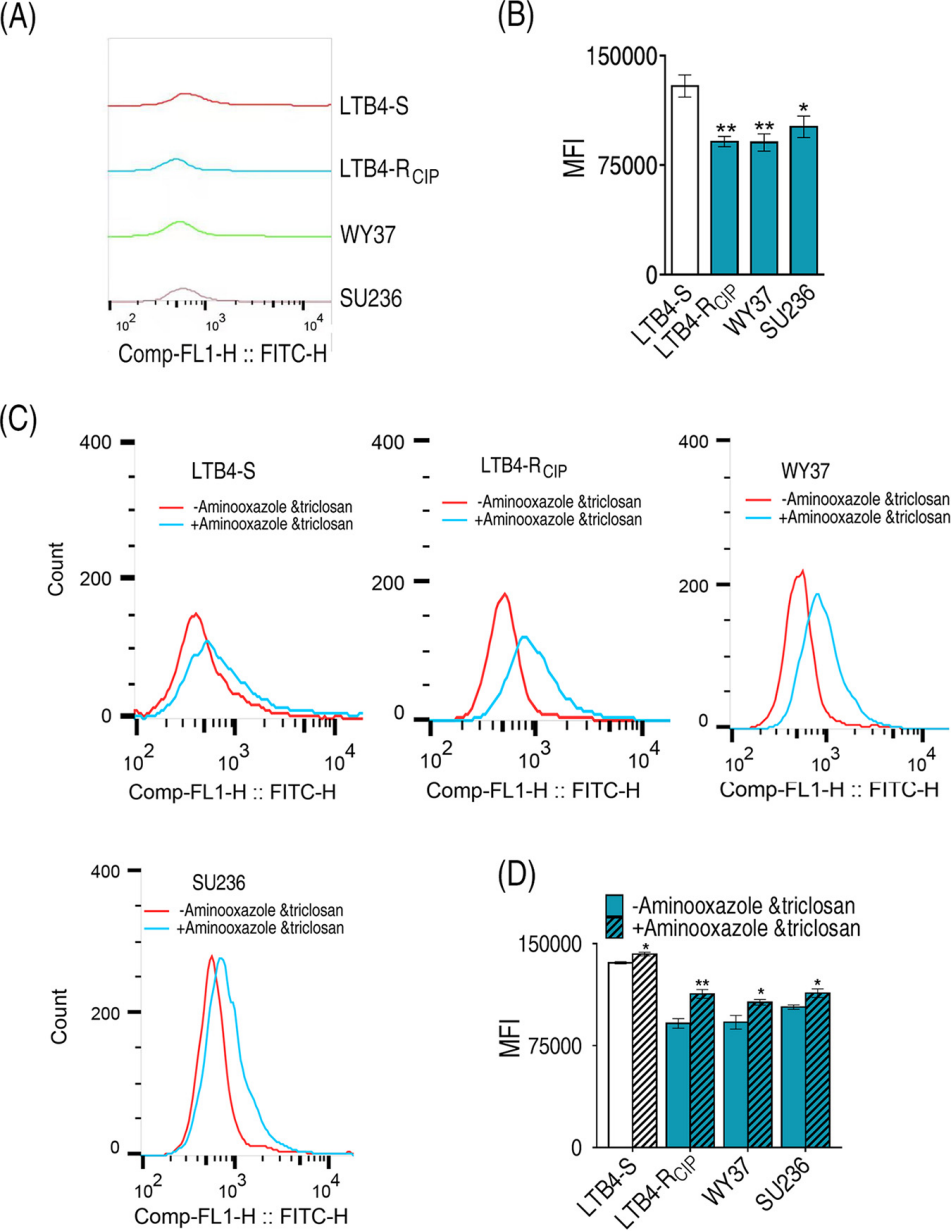
Membrane permeability among *E. tarda* strains. (A) Single-parameter histogram. (B) Green fluorescence signal intensity. (C) Single-parameter histogram in the presence or absence of 2-aminooxazole and triclosan. (D) Green fluorescence signal intensity in the presence or absence of 2-aminooxazole and triclosan. Results in panels B and D are displayed as mean ± SEM, and significant differences are identified: *, *P* < 0.05, and **, *P* < 0.01, by two-tailed Student's *t* test.

The incorporation of fatty acids affects membrane permeability (40). Therefore, membrane permeability was measured in LTB4-R_CIP_, WY37, SU236, and LTB4-S. LTB4-S had much higher membrane permeability than LTB4-R_CIP_, WY37, and SU236 (Fig. 8A and B). Further comparison was performed of the four strains with and without 2-aminooxazole and triclosan. The two inhibitors caused the fluorescence peaks to shift to the right, leading to higher membrane permeability in the four strains (Fig. 8C and D). These results indicate that the reduction of membrane permeability is a mechanism by which *E. tarda* resists antibiotics, supporting the conclusion that fatty acids are related to membrane permeability.

## DISCUSSION

The present study adopted quantitative proteomics to explore the mechanism of *E. tarda* resistance to ciprofloxacin. The ciprofloxacin-resistant proteome consisted of 233 differential proteins, including 142 upregulated proteins and 91 downregulated proteins. Among the altered proteins, 53 proteins functionally belonged to metabolism. KEGG pathway analysis showed that the upregulated proteins and downregulated proteins affected eight and six metabolic pathways, respectively, where 15 genes (Rbsk, RibB, RibH, KbaZ, GatY, AgaV, FbaA, GpmA, PyrF, AcnB, SucB, SucC, SucD, SdhC, and Mdh) belong to the central carbon metabolism and energy metabolism. Among the nine enriched biological functional categories in the protein-protein interaction, six pathways (galactose metabolism, TCA cycle, oxidative phosphorylation, riboflavin metabolism, glycine, serine and threonine metabolism, and purine metabolism) are for metabolism. Recently, proteomics-based ciprofloxacin resistance mechanisms have been reported in other genera but were not involved in *E. tarda* (26). Ciprofloxacin resistance develops from a low to high level in Pseudomonas aeruginosa, in which two different low levels and one high level of resistant molecular mechanisms have been identified. Among the two low-level mechanisms, one is the switch from anaerobic respiration by increased expression of catalase and peroxidase, and the other is probably due to iron, polyamine, and DNA repair. When the high level of resistance is obtained, major players include the efflux pump MexCD-OprJ and the downregulation of quorum sensing (QS) (41). The adaptive response of P. aeruginosa biofilms to supra-MIC treatment with ciprofloxacin includes the upregulation of proteins for sensing and repairing DNA damage and substantial changes in the expression of enzymes involved in central carbon metabolism (42). Continuous adjustment of metabolic patterns to enhance nucleotide synthesis, energy generation, and QS genes in the early growth phase is a characteristic feature in Escherichia coli in resistance to ciprofloxacin (43). However, the response of the central carbon metabolism has not been determined in ciprofloxacin resistance. The present study indicates that the P cycle is disrupted in ciprofloxacin-resistant *E. tarda*, suggesting that the inactivation of the P cycle is required for antibiotic resistance. This finding is consistent with recent reports that the depressed central carbon and energy metabolisms are associated with the acquisition of antibiotic resistance in bacteria (31, 35, 44–47). Therefore, the central carbon metabolism plays a key role in antibiotic resistance.

Both proteomics and gene expression data indicate that the pyruvate cycle, a center of the central metabolism (48), is impaired in antibiotic-resistant bacteria. Actually, the activity of TCA, being part of the pyruvate cycle, was decreased in different types of antibiotic-resistant bacteria (31, 49). However, the effects of the pyruvate cycle on antibiotic-resistant bacteria were largely unexplored. In this study, we found that the expression of genes and the expression of proteins were not well correlated. The transcription of genes encoding the enzymes that drive the metabolic flux of succinate to oxaloacetate and from PEP to pyruvate were increased, while the protein abundance of these enzymes was decreased, whereas genes encoding enzymes that mediate the flux of pyruvate to succinate were decreased. This discrepancy was also observed when both the protein-protein interaction network and iPath analysis were applied. Therefore, functional studies were performed to validate the increase or decrease of the pyruvate cycle. As an example of the analysis, the expression of AcnB was increased as identified by proteomics, while the transcriptional level was reduced. In addition, the reduction of purine biosynthesis also provides proof of the disrupted P cycle in LTB4-R_CIP_ since the P cycle provides KGDH for transformation of glutamic acid and then glutamine for purine biosynthesis. That the difference between protein abundance and gene expression does not correlate was not astonishing as posttranscriptional regulation plays a role (50). However, our study actually presents a possible complex network that governs antibiotic resistance more than metabolism and proteomics. Our results together indicate that the P cycle was disrupted, and thus energy was reduced in LTB4-R_CIP_.

iPath analysis indicates increased lipid metabolism, suggesting that a metabolic flux enters into biosynthesis of fatty acids. To test this, qRT-PCR and enzymatic activity were used to quantify the gene expression and enzyme activity of the fatty acid biosynthesis. The elevation of most gene expression and ACC activity validates the increased fatty acid biosynthesis. ACC catalyzes the first rate-limiting step in the fatty acid biosynthesis pathway through formation of malonyl-CoA from acetyl-CoA, thereby providing solid proof for the increased fatty acid biosynthesis. Further investigation focused on whether the activated biosynthesis of fatty acids contributes to ciprofloxacin resistance. When the inhibitors 2-aminooxazole and triclosan are used to inhibit ACC and FabF to reduce acetyl-CoA into biosynthesis of fatty acids and acyl-ACP into biosynthesis of saturated fatty acids, respectively, ciprofloxacin-mediated killing is increased. Thus, the biosynthesis of fatty acids positively accounts for the resistance. Both 2-aminooxazole and triclosan are antibacterial chemicals. There is a correlation between decreased susceptibility to triclosan and ciprofloxacin resistance and acyl carrier protein (ACP) reductase as a target for antibacterial drugs (37, 38), but the association of 2-aminooxazole with ciprofloxacin resistance is unknown. Our results not only indicate that the elevated biosynthesis of fatty acids plays a role in ciprofloxacin resistance, but also show that the synergistic use of 2-amonooxazole or/and triclosan and quinolone promotes quinolone-mediated killing. Furthermore, the approach is effective with clinically isolated antibiotic-resistant *E. tarda* strains, thereby providing a way to eliminate these clinical strains by quinolone antibiotics. In addition, the present study identifies a correlation between elevated fatty acid biosynthesis and ciprofloxacin resistance, but the fatty acids that actually play a role should be further characterized. However, our results show that inhibition of the metabolic flux into biosynthesis of fatty acids by 2-aminooxazole or biosynthesis of saturated fatty acids by triclosan elevates bacterial sensitivity to ciprofloxacin. These results suggest that ratio of the saturated and unsaturated fatty acids may account for the antibiotic resistance.

Fatty acids contribute to bacterial membrane permeability. Therefore, differences in bacterial membrane permeability are measured between antibiotic-sensitive and -resistant *E. tarda* strains and in the presence or absence of 2-amonooxazole and triclosan by using SYTO-9 green fluorescent dye. Our results show that lower membrane permeability is detected in antibiotic-sensitive than -resistant *E. tarda*. When the two inhibitors are used, the membrane permeability is increased. Thus, the incorporation of fatty acids variably affects how membrane permeability contributes to the antibiotic resistance, but the exact mechanisms await investigation.

Besides biosynthesis of fatty acids, the P cycle, and energy metabolism, the present study also shows that the ABC transporter, homologous recombination, and ribosomes may play roles in ciprofloxacin resistance. Among the pathways, the ABC transporter and homologous recombination are reported in ciprofloxacin resistance. However, the role of ribosome in the ciprofloxacin resistance is largely unknown. Ciprofloxacin is a bactericidal antibiotic that does not target ribosomes (51). In the use of ciprofloxacin to combat staphylococcal infections, 70S ribosome levels are reduced, since ciprofloxacin is known for its inhibitory effects on the bacterial ribosome (52). There is no relationship between the susceptibility of E. coli strains with fewer ribosomal operons and killing by ciprofloxacin (53). The present study shows elevated levels of ribosomes RpmL, RpsM, RpmD, RpsT, RpsN, and RplM in LTB4-R_CIP_ and the interaction of these ribosomes with the TCA cycle, purine biosynthesis, and homologous recombination. Therefore, these findings on the elevated expression of ribosomes and their interaction provide a clue to further understand ciprofloxacin resistance. At last, we have to acknowledge that the proteomic change of bacteria upon obtaining antibiotic resistance may directly contribute to ciprofloxacin resistance, which may indirectly influence the metabolism. Thus, the proposed effects on membrane functionality to limit transport of the quinolone may have collateral effects on uptake of nutrients, indicating the complex interplay between bacteria, antibiotics, and the environment. Additionally, whole-genome sequencing to probe the genetic mutations and whether such mutations affect metabolism are worthy of further investigation.

### Conclusion.

**FIG 9:**
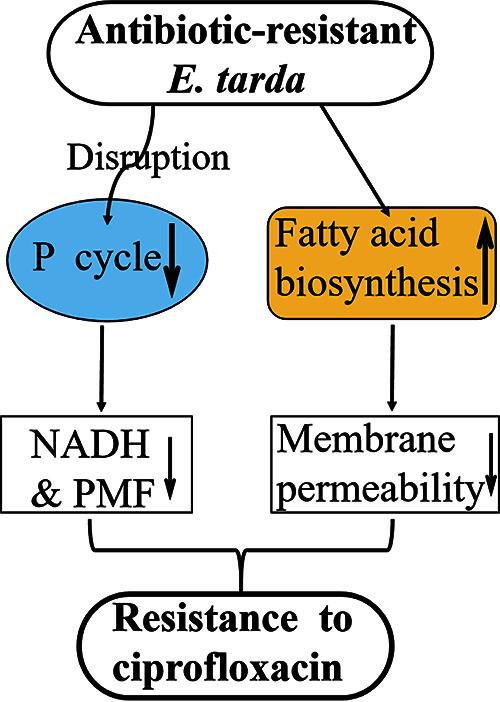
Mechanism of ciprofloxacin resistance in *E. tarda.* In the ciprofloxacin-resistant *E. tarda* LTB4 strain (LTB4-R_CIP_), the pyruvate cycle is reduced, leading to a decreased concentration of NADH and membrane potential. On the other hand, biosynthesis of fatty acids is enhanced while membrane permeability is reduced, which promotes bacterial insensitivity to ciprofloxacin.

The present study aims to explore the bacterial response to ciprofloxacin at the proteome level. Our results demonstrate that the ciprofloxacin resistance affects multiple layers of cellular activities, where the P cycle, energy metabolism, and biosynthesis of fatty acids play a key role in the resistance. Specifically, the P cycle, which connects glycolysis to glutamate metabolism and then purine biosynthesis, is reduced. Therefore, NADH, membrane potential, ATP, and purine biosynthesis are decreased. On the other hand, biosynthesis of fatty acids is enhanced and thereby promotes bacterial insensitivity to ciprofloxacin, which is related to membrane permeability (Fig. 9). These findings deepen the understanding of ciprofloxacin resistance.

## MATERIALS AND METHODS

### Bacterial strains.

The bacterial strains used in this study included the ciprofloxacin-resistant LTB4 strain (LTB4-R_CIP_) and its parent strain, *E. tarda* LTB4 (LTB4-S). LTB4 was obtained from Xiaohua Zhang, Ocean University of China. LTB4-R_CIP_ (16-fold MIC, 2.5 μg/ml) was selected from LTB4 through continuous propagation in Luria-Bertani (LB) medium plus 1/2 MIC of ciprofloxacin (0.078 μg/ml). The LTB4 strain serving as a control was cultured in the same medium without ciprofloxacin and named ciprofloxacin-sensitive *E. tarda* LTB4-S. A single colony was cultured in LB medium at 30°C for 24 h, then diluted at 1:100, and grown to an optical density at 600 nm (OD_600_) of 1.0. The pellet was collected for functional analysis by centrifugation at 8,000 × *g* for 5 min.

This article does not contain any studies with human participants or animals performed by any of the authors.

### MIC determination.

The MIC was determined by the microdilution method as previously described (54). Briefly, ciprofloxacin was 2-fold serially diluted with LB medium. The concentrations of ciprofloxacin were set ranging from 0.078 to 5 μg/ml (0.078, 0.156, 0.312, 0.625, 1.25, 2.5, and 5 μg/ml), which complies with the law of 2-fold dilution. Ten microliters of 10^5^ CFU of logarithmic-phase bacteria and 90 μl of LB medium were added to a 96-well plate, which was cultured for 24 h. The lowest concentration observed without visible growth was recorded as 1 MIC. At least three biological replicates were performed.

### Protein sample preparation and iTRAQ labeling.

The protein sample preparation and iTRAQ labeling were performed as previously described (55). In brief, the two strains LTB4-S and LTB4-R_CIP_ were cultured in LB medium without ciprofloxacin and grown at 30°C for 24 h. Cells were inoculated (1:100 dilution) in new 50 ml LB in 250-ml baffled flasks at 30°C and 200 rpm until the OD_600_ reached 1.0. Cells were collected and washed. Then, 100 μg protein was quantified from each group, dissolved in 200 mM Tris(2-carboxyethyl)phosphine hydrochloride (TCEP), incubated at room temperature for 1 h, and then mixed with 25 mM iodoacetamide (IAA), which alkylated in the dark for 1 h. Furthermore, the mixtures were digested with trypsin (Promega, USA) overnight at 37°C at a ratio of 1:50. The digested peptides were labeled with an iTRAQ labeling kit (AB Sciex, Canada), and the proteomics analysis was carried out with two biological replicates. The labeled peptides were mixed and desalted with a Strata X C_18_ desalting column (Phenomenex, USA). The 8 fractions were separated by reverse-phase high-performance liquid chromatography (RP-HPLC) and then identified using mass spectrometry.

### Proteomics analysis.

Liquid chromatography-tandem mass spectrometry (LC-MS/MS) was performed in a NanoAcquity UPLC (ultraperformance liquid chromatography) device (Waters, USA) coupled with an AB SciexTripleTOF 5600 mass spectrometer (AB Sciex, Canada). The acquisition of MS data was performed the same as previously described (56). Peptide spectrum matching of MS/MS spectra was performed against the *E. tarda* database by ProteinPilot 4.5 software (Applied Biosystems, USA), and the search parameters included immobilized modification to carbamidomethyl cysteine and variable modification to methionine oxidation, as well as iTRAQ reagent labeling at the N terminus of peptides and trypsin digestion to reduce missed cleavage. A relatively conservative threshold (false discovery rate of <1%, with at least one unique peptide matched) was used for protein quantification and confidence. The identification of differentially expressed proteins was based on fold change ratio of ≥1.5 or ≤0.67 at *P* values of <0.05. IBM SPSS statistics 19 software was used to analyze the correlation between the two biological replicates. The OmicsBean website (http://www.omicsbean.cn/) was used to analyze Gene Ontology (GO) annotations and Kyoto Encyclopedia of Genes and Genomes (KEGG) pathways of the differential expression of proteins. The functions of proteins were classified by the biological process hierarchical clustering of GO annotation. This was used to analyze the protein-protein interaction network by the retrieval of interacting genes/proteins (STRING) (57). Finally, the network tool interactive pathway explorer iPath (http://pathways.embl.de) was used to visualize and analyze cellular pathways (58).

### Measurement of enzyme activity.

Enzyme activities of pyruvate dehydrogenase (PDH), α-ketoglutarate dehydrogenase (α-KGDH), succinate dehydrogenase (SDH), and malate dehydrogenase (MDH) were quantified as described above (59). In brief, 30-ml bacterial samples with an OD_600_ of 1.0 were collected. These bacteria were washed with sterile saline (0.85%) by centrifugation and then resuspend in sterile saline, sonicated on ice (with power set to 200 W) for 5 min, and then centrifuged at 4°C for 5 min at 8,000 rpm. Supernatants were transferred to a new tube, and the amounts of protein were determined by Bradford assay. The following reaction mixture was used for detection of enzyme activity: 2.5 mM MgCl_2_, 0.15 mM MTT [3-(4,5-dimethyl-2-thiazolyl)-2,5-diphenyl-2H-tetrazolium bromide], 0.2 mM thiamine PP_i_ (TPP), 0.5 mM phenazine methosulfate (PMS), and 50 mM potassium phosphate buffer (pH 7). To this mixture, 5 mM substrate (pyruvate, α-ketoglutarate, succinate, or malate) was then added. The 200 μg of quantified total protein was incubated at 37°C with the reaction mixture for 30 min. Enzyme activities were quantitatively measured by spectrophotometer to monitor the reduction of MTT using a Victor X5 multimode microplate reader at 566 nm. At least three biological replicates were measured for each enzyme assay. In addition, acetyl-CoA carboxylase (ACC) was measured with a commercially available ACC activity assay kit (Solarbio Life Science, China, catalog no. BC0415) according to the manufacturer’s manual.

### Membrane potential measurement.

Membrane potential was measured using the BacLight bacterial membrane potential kit (Invitrogen, USA) as previously described (60, 61). First, 1 ml of bacterial suspension at an OD_600_ of 1.0 was diluted to obtain 10^6^ CFU/ml with sterile saline, and then 30 μM DiOC_2_(3) (3,3′-diethyloxyfluorocarbon iodide) reagent was added for incubation. The mixture was incubated at 30°C and shaken at 200 rpm for 30 min. Finally, the samples were analyzed by flow cytometry (FACSCalibur, USA). DiOC_2_(3) was excited at 488 nm; its green fluorescence and red fluorescence were detected by 530- and 610-nm band-pass filters, respectively. The size and membrane potential depend on the intensity of red fluorescence. The calculation of membrane potential was based on the red/green fluorescence intensity ratios, following the formula log of [10^3/2^ × (red fluorescence/green fluorescence)].

### Quantification of NADH content.

NADH was quantified according to the EnzyChrom NAD/NADH assay kit (Bioassay Systems, USA). One milliliter (OD_600_ of 1.0) of bacterial suspension was collected, resuspended in NADH extraction buffer, and incubated in a 60°C water bath for 5 min. The reaction mixtures were then neutralized with neutralization buffer, briefly vortexed, and centrifuged at 14,000 rpm for 5 min. The resulting supernatant was used to measure the NADH concentration. The relative amount of NADH was determined by comparison to a known concentration of NAD^+^ standard curves. The optical densities at time zero (OD0) and after a 15-min incubation (OD15) were read at 565 nm (from 520 to 600 nm) at room temperature. The OD0 values were subtracted from the OD15 for standard and sample concentration analysis. The ΔOD values were used to determine the sample concentration of NADH from the standard curve.

### Quantitative reverse transcription-PCR.

Quantitative reverse transcription-PCR (qRT-PCR) was performed as previously described (62). In brief, total RNA was extracted from 1 ml of bacterial suspension at an OD_600_ of 1.0. Total RNA was extracted from each sample with TRIzol (Invitrogen, USA). Reverse transcription of cDNAs with 1 μg of RNA was performed with a PrimeScript RT reagent kit with gDNA Eraser (TaKaRa, Japan). PCR was carried out in 10 μl of the total reaction mixture, containing 5 μl of 2× SYBR Premix *Ex Taq* (TaKaRa, Japan), 1 μl of cDNA template, 0.2 μl of 10 μM forward and reverse primers, and 3.6 μl sterile water. The sequences of primers are listed in Tables S5 and S6 in the supplemental material. Quantification of mRNA expression was detected on the LightCycler 480 system (Roche, Germany). The cycling conditions were set as follows: denaturation for 30 s at 95°C to activate polymerase, followed by 40 cycles of 95°C for 5 s and 57°C for 30 s. Fluorescence measurements were measured for each cycle to 70°C for 1 s. The amplification program was terminated by each cycle of 95°C and with a heating rate of 5°C/s for melting curve acquisition. For analysis of relative gene expression, data were converted to a percentage of the value of the control group. The highly conserved region of the 16S rRNA gene in bacteria was used as an internal control. At least triplicate repeats were carried out. All assays were performed in triplicate and repeated at least three times.

10.1128/mSystems.00694-21.7 TABLE S5 Primers used for qRT-PCR about the P cycle gene. Download Table S5, XLS file, 0.03 MB. Copyright © 2021 Su et al. 2021 Su et al. https://creativecommons.org/licenses/by/4.0/ This content is distributed under the terms of the Creative Commons Attribution 4.0 International license.

10.1128/mSystems.00694-21.8 TABLE S6 Primers used for qRT-PCR about the fatty acid biosynthesis gene. Download Table S6, XLS file, 0.03 MB. Copyright © 2021 Su et al. 2021 Su et al. https://creativecommons.org/licenses/by/4.0/ This content is distributed under the terms of the Creative Commons Attribution 4.0 International license.

### Measurement of ATP content.

ATP content was measured as previously reported (63). Briefly, bacterial cells were grown to an OD_600_ of 1.0, and 1 ml of cell culture was collected, diluted with saline to obtain a concentration of 10^7^ CFU/ml, and distributed to a 96-well plate. An equal volume of BacTiter-Glo assay reagent (Promega, USA) was added to the plate per the manufacturer’s instruction. Luminescence was measured by the Victor X5 multimode microplate reader. The luminescence values corresponding to the same volume as 0, 10^1^, 10^2^, 10^3^, 10^4^, 10^5^, and 10^6^ pmol/liter ATP were determined to establish a linear relationship between luminescence value and ATP concentration and luminescence ratio values. Intracellular ATP levels of LTB4-S and LTB4-R_CIP_ were calculated according to this relationship.

### Antibiotic bactericidal assay.

The antibiotic bactericidal assay was carried out as previously described (61). In brief, well-cultured bacterial cells were collected, washed 3 times with saline, and then resuspended in M9 medium to an OD_600_ of 0.2. The cells were diluted to 1:100 using M9 medium. An aliquot of 5 ml was tested in addition to the desired antibiotics and inhibitor and incubated at 30°C at 200 rpm for 6 h. To determine CFU per milliliter, 100 μl of samples was removed and serially diluted, and an aliquot of 10 μl of each dilution was spot-plated onto LB agar plates and cultured at 30°C. Only dilutions that yielded 20 to 200 colonies were available. The percentage of survival was determined by dividing the CFU obtained from a treated sample by the CFU obtained from control.

### Membrane permeability measurement.

Measurement of bacterial cell membrane permeability was performed as previously described (65). Briefly, 1 ml of bacterial cells was collected by centrifugation and resuspended in an equal volume of phosphate-buffered saline (PBS) diluted to a concentration of 10^6^ CFU/ml. One milliliter of the cell suspension was mixed with 2 μl SYTO-9 green fluorescent dye (Invitrogen, USA) to a final concentration of 50 μM and incubated for 15 min (or 5 min for LTB4-S) at 30°C. All samples were immediately transferred to the flow cytometry analysis tube and detected by flow cytometry to obtain the green fluorescence intensity. At least three independent experiments were performed.

### Growth curve measurement.

A single clone of *E. tarda* was cultured in LB medium for 24 h to reach saturation. Then, the culture was inoculated to 50 ml of LB broth in 250-ml flasks at a ratio of 1:100 (vol/vol) and incubated at 30°C in a shaker at 200 rpm. Samples were taken and measured once every 2 h for a total of 24 h. The OD_600_ values were recorded. Three biological replicates were carried out.

### Data availability.

The iTRAQ-based proteomics data are available in FigShare (https://doi.org/10.6084/m9.figshare.14610129).

10.1128/mSystems.00694-21.9 TABLE S7 Primers used for PCR of the *gyrA* gene. Download Table S7, XLS file, 0.02 MB. Copyright © 2021 Su et al. 2021 Su et al. https://creativecommons.org/licenses/by/4.0/ This content is distributed under the terms of the Creative Commons Attribution 4.0 International license.
