# Supplementary figures and images for: Enhanced Biosynthesis of Fatty Acids Is Associated with the Acquisition of Ciprofloxacin Resistance in Edwardsiella tarda
Source: mSystems. 2021 Aug 24;6(4):e00694-21. doi: 10.1128/mSystems.00694-21 (PMC8407472; doi:10.1128/mSystems.00694-21)

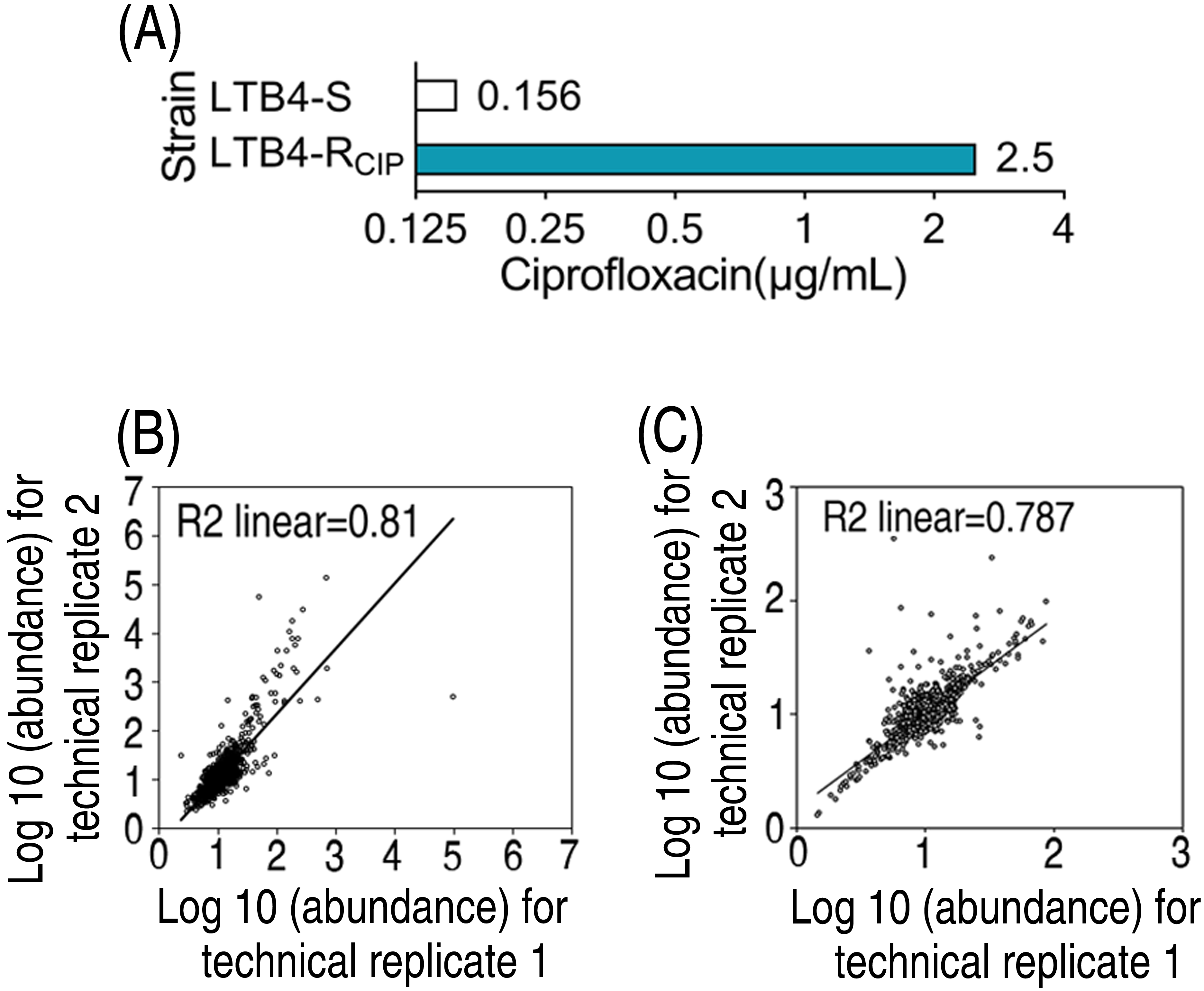

Supplement: FIG S1 [file msystems.00694-21-sf001.tif]

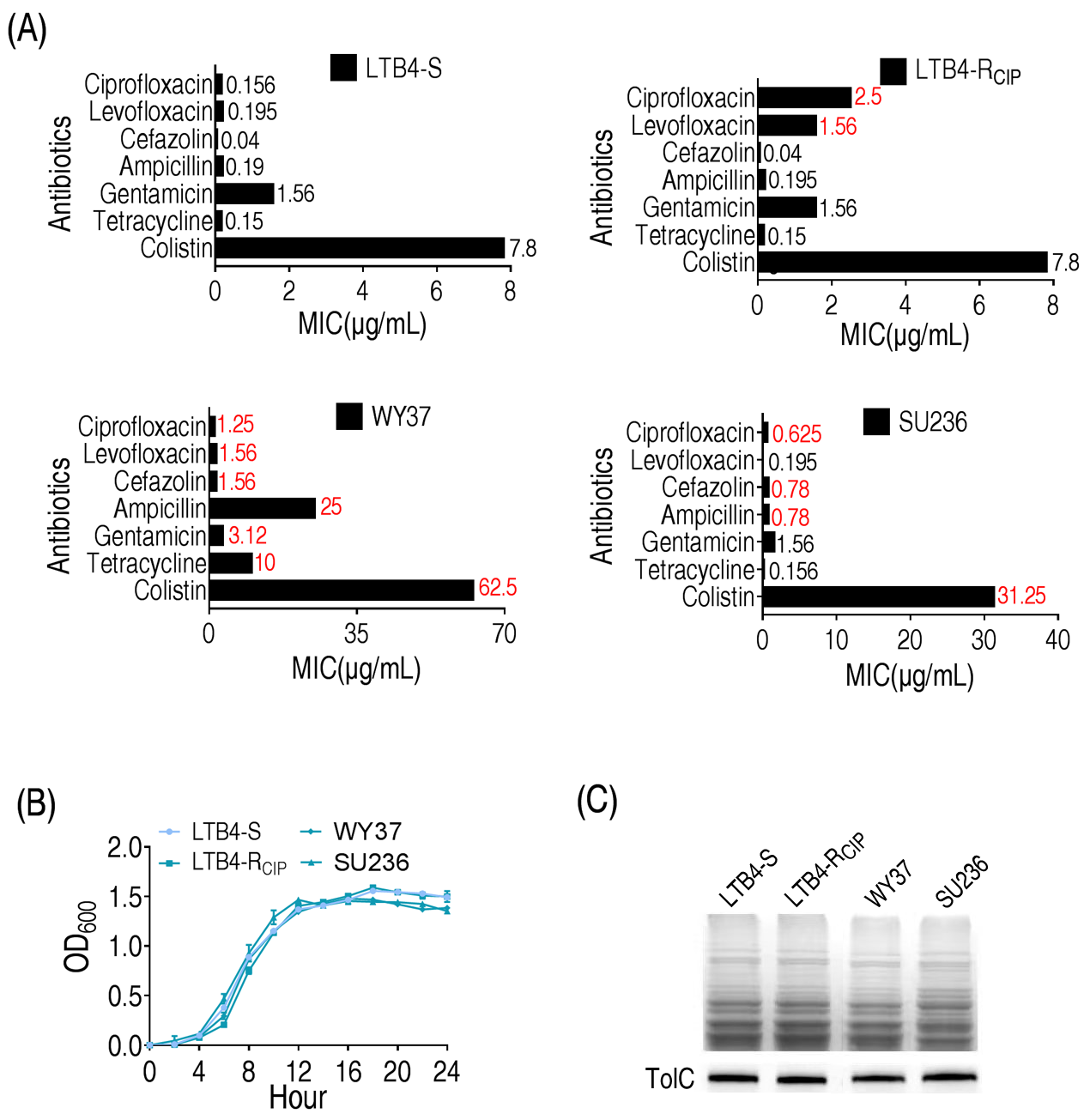

Supplement: FIG S2 [file msystems.00694-21-sf002.tif]
